# Urea-based mutualistic transfer of nitrogen in biological soil crusts

**DOI:** 10.1093/ismejo/wrae246

**Published:** 2024-12-13

**Authors:** Ana Mercedes Heredia-Velásquez, Soumyadev Sarkar, Finlay Warsop Thomas, Ariadna Cairó Baza, Ferran Garcia-Pichel

**Affiliations:** Center for Fundamental and Applied Microbiomics, Biodesign Institute, Arizona State University, Tempe, AZ 85287, United States; School of Life Sciences, Arizona State University, Tempe, AZ 85287, United States; Center for Fundamental and Applied Microbiomics, Biodesign Institute, Arizona State University, Tempe, AZ 85287, United States; Center for Fundamental and Applied Microbiomics, Biodesign Institute, Arizona State University, Tempe, AZ 85287, United States; School of Life Sciences, Arizona State University, Tempe, AZ 85287, United States; Center for Fundamental and Applied Microbiomics, Biodesign Institute, Arizona State University, Tempe, AZ 85287, United States; Center for Fundamental and Applied Microbiomics, Biodesign Institute, Arizona State University, Tempe, AZ 85287, United States; School of Life Sciences, Arizona State University, Tempe, AZ 85287, United States

**Keywords:** biocrusts, *Microcoleus vaginatus*, *Massilia*, *Arthrobacter*, *Bacillus*, cyanobacteria, diazotroph, symbiosis, nitrogen, urea, cyanosphere

## Abstract

Foundational to the establishment and recovery of biocrusts is a mutualistic exchange of carbon for nitrogen between pioneer cyanobacteria, including the widespread *Microcoleus vaginatus*, and heterotrophic diazotrophs in its "cyanosphere". In other such mutualisms, nitrogen is transferred as amino acids or ammonium, preventing losses through specialized structures, cell apposition or intracellularity. Yet, in the biocrust symbiosis relative proximity achieved through chemotaxis optimizes the exchange. We posited that further partner specificity may stem from using an unusual nitrogen vehicle, urea. We show that representative mutualist *M. vaginatus* PCC 9802 possesses genes for urea uptake, two ureolytic systems, and the urea cycle, overexpressing only uptake and the rare urea carboxylase/allophanate hydrolase (uc/ah) when in co-culture with mutualist *Massilia* sp. METH4. In turn, it overexpresses urea biosynthesis, but neither urease nor urea uptake when in co-culture*.* On nitrogen-free medium, three cyanosphere isolates release urea in co-culture with *M. vaginatus* but not in monoculture. Conversely, *M. vaginatus* PCC 9802 grows on urea down to the low micromolar range. In natural biocrusts, urea is at low and stable concentrations that do not support the growth of most local bacteria, but aggregates of mutualists constitute dynamic microscale urea hotspots, and the cyanobacterium responds chemotactically to urea. The coordinated gene co-regulation, physiology of cultured mutualists, distribution of urea pools in nature, and responses of native microbial populations, all suggest that low-concentration urea is likely the main vehicle for interspecies N transfer, helping attain partner specificity, for which the rare high-affinity uc/ah system of *Microcoleus vaginatus* is likely central.

## Introduction

Biological soil crusts, or biocrusts, are topsoil photosynthetic, largely microbial communities encompassing bacteria [1], fungi [2], archaea [3], and microalgae [4], that may develop to include lichens [5] and mosses [6]. They are globally distributed, covering up to 12% of the Earth's terrestrial surface and 30% of all dryland soils [7], where they become a prominent player in ecosystem processes, providing ecosystem services [8] that include soil stabilization against erosion and the fertilization of the soils they cover. The development of biocrusts is based on the primary productivity of their photosynthetic members, which define different biocrust types and an ecological succession framework to explain their transition from incipient cyanobacterial biocrusts to complex cyanobacterial biocrusts, and to eventually include lichen and moss populations (reviewed in [9]). Only some cyanobacteria can initiate biocrust formation; all are filamentous, non-heterocystous types that form bundles of trichomes held within a common sheath. These include several genera in the *Coleofasciculaceae* and *M. vaginatus* in the *Microcolaceae* [10]. The latter is the most common and abundant among them, and perhaps the most abundant terrestrial cyanobacterium globally [11]. The evolutionary convergent capacity to form bundles is regarded as key to stabilization of unconsolidated soils on contact [12]. Thus, *M. vaginatus* plays a key pioneering role in biocrust ecological succession and in biocrust recovery on degraded soils.

Whereas *M. vaginatus* displays a variety of unique adaptations [13–15] in line with the pulsed regime of water availability, harsh conditions and low nutrients typical of arid topsoils, one trait had until recently remained paradoxical: *M. vaginatus* cannot fix nitrogen [16], even when biocrusts are in a perennial state of nitrogen limitation [17]. Instead, it relies on mutualistic C for N exchanges with a subset of heterotrophic biocrust bacteria. These are preferentially recruited from the bulk biocrust microbiome, conforming a cyanosphere highly enriched in diazotrophs that resides on a bundle’s confining EPS sheath [18]. The mutualism has been successfully reproduced in vitro using representative partner cultures [19], allowing the growth of co-cultures with CO_2_ and N_2_ as sole sources of C and N for growth. The provision of mutualistic heterotrophs (“biocrust probiotics”) in inoculum used for soil restoration leads to more robust biocrust development than the provision of the cyanobacterium alone [20, 21].

In nature, mutualisms based on the exchange of metabolites exist on a continuum involving ephemeral spatial co-location of partners [22] to highly integrated endosymbioses [23]. Confinement into either host structures or intracellular settings allows for precise partner control and specificity. The root nodules of rhizobial bacteria, monitored for cheaters by the plant hosts [24, 25] are an example. Confinement also serves to limit exometabolite losses to neighboring, non-mutualistic bacteria, as with the intraradical hyphal nets of ectomycorrhizal fungi [26, 27]. The *M. vaginatus* / cyanosphere mutualism, by contrast, sits at the opposite end of this continuum, involving no dedicated structures, and apparently relying on simple spatial proximity between partners. Spatial co-location at the microscale is optimized through the cyanobacterium’s motility behavior in response to specific chemical cues by the heterotrophs, and amplified by the cyanobacterium’s own quorum sensing, a communication enabled by GABA and Glutamate signals [28]. It is yet unknown if the heterotrophs also respond to cues from the cyanobacterium, though at least some mutualists are non-motile.

Optimized co-location, however, still leaves extracellular metabolite exchanges principally vulnerable to exploitation by adventitious non-mutualists. Hence, we postulated that additional mechanisms might exist to attain transfer specificity between partners, perhaps involving the nature of the metabolites exchanged. In well studied mutualisms where N is exchanged, carriers are typically ammonium [29–31], amino acids (Glu, Gln, and Arg; [32–34]), or both [35]. In our case, ammonium seems like an unlikely candidate because competitive losses to the large ammonia-oxidizing archaeal and bacterial populations typical of biocrusts [36] should be expected. Typical amino acid N-carriers are also unlikely because, in culture, *M. vaginatus* grows only poorly on Gln and Arg as sole source of N, and not at all on Glu. Its preferred N source is urea [28], even when most bacteria prefer ammonium [37]. We therefore examined the role of urea as a potential N transfer molecule in our mutualistic system more closely, using both representative isolates of the partners and native *M. vaginatus* bundles inclusive of their attached cyanospheres, with interrogations at the ecological, physiological and molecular biological level.

## Materials and methods

### Sampling, strains, and culture conditions

Intact biocrust samples were collected from sites previously described at Poly Ground Mount 2, Mesa, Arizona, and the Jornada Experimental Range, Las Cruces, New Mexico, using Petri plates and comprising the top 1 cm of soil. *M. vaginatus* was known to be the dominant cyanobacterium in these biocrusts [20, 21]. Axenic cyanobacterial cultures of *M. vaginatus* PCC 9802 and *Synechocystis* sp*.* PCC 6803 were maintained in 50% BG11 medium [38]. *Nostoc punctiforme* ATCC 29133 was maintained in 50% N-free BG11_0_ medium. Cyanobacteria were incubated at 23°C with 18–20 μE m^−2^ s^−1^ of white light illumination under a 14 h/ 10 h dark cycle. BG11 medium was amended as needed for subsequent work. Since these cyanobacterial strains have been kept in culture for a long time, natural samples of mutualists were used as confirmation for the culture-based findings whenever feasible. The heterotrophic mutualists *Arthrobacter* sp. O80, *Bacillus* sp. O64, and *Massilia* sp. METH4, originally isolated from the cyanosphere of *M. vaginatus* [38], were maintained in N-free Burks medium [39] at 23°C without illumination.

### Bundle migration assays

To examine bundle migration under various conditions, nitrogen sources (ammonium, nitrate, and urea) were added in solution at concentrations of 10 mM and 100 μM. Native biocrusts were crumbled into mm-size thin pieces and placed on Petri plates filled with native soil collected 5 cm under the crust. The soil was then wetted to saturation using appropriate aqueous test solutions in sterile 18 μΩ Milli-Q water, or a control solution of sterile 18 μΩ Milli-Q water, and incubated for 24 h under standard cultivation conditions as above, after which we counted the number of bundles migrating out of the initial inoculum and into the soil under a dissecting microscope (n = 5 biocrust pieces per treatment).

### Urea and ammonium determinations

Urea was determined colorimetrically by an enzyme-coupled reaction using a commercial kit (Amplite Colorimetric Urea Quantitation Kit ^*^Blue Color^*^), following the manufacturer’s instructions. Its detection limit varied between 2 and 5 μM depending on specific runs. Ammonium was also determined with a colorimetric kit (Amplite Colorimetric Ammonia Quantitation Kit ^*^Blue color^*^) with an effective detection limit around 5 μM.

For measurements in the soil solution, samples were wetted to saturation with sterile 18 μΩ Milli-Q water, incubated for the appropriate times as reported, then placed on a Nalgene filter unit with a polyethersulfone membrane of 0.2 μm pore size. The soil solutions were then vacuum-filtered and collected prior to analyses.

For measurements in liquid cultures, 8 ml independent cultures were incubated for 5 days. Gravity-settled media was then collected and analyzed directly. Concurrently, biomass dry weight was determined gravimetrically after vacuum-filtration of culture aliquots on pre-weighed 0.20 μm pore size Sterlitech polycarbonate filters; the samples were then left to dry at ~13% humidity and room temperature to obtain biomass-specific rates of extracellular release. To obtain biomass-specific rates of release in co-cultures of the cyanobacterium with heterotrophs, total dry mass of both members was determined as above, and then Chl *a* extracted and determined spectrophotometrically [40], which was used to estimate the biomass of cyanobacterium in the mixture (Chl *a* is ~0.97% of DW in *M. vaginatus* PCC 9802 under our culture conditions), and the biomass of the heterotrophs estimated by difference from totals. The fraction of heterotrophic biomass was comparable in all replicates, though variable among strains. Typically, it would consist of around 35% of the total biomass.

To estimate extracellular urea around native *Microcoleus* / cyanosphere bundles, >300 bundles were excised from biocrusts by micromanipulation, using forceps under a microscope [18]. These were collected in multiwell plates in a dry state until experimentation. After adding 200 μl of distilled water to each collection, they were incubated under standard phototroph conditions (above) for either 3 or 24 h. These incubation times were selected to ensure that the microbes were active and to resemble a long rain event, respectively. Urea in solutions was determined in aliquots with the standard assay. For biomass normalization, each bundle was photographed under the dissecting scope, its diameter and length measured by image analysis using Image J [41], and its biovolume computed assuming a cylindrical shape. From this data total biovolumes in each bundle collection were computed, and urea concentrations corrected for differences in total biovolume by normalizing to a common ratio (incubation volume to biovolume) of 1000.

Intracellular urea was determined from dry pellets of monocultures or co-cultures (*n* = 2 independent cultures) and also from bundle collections after sample resuspension in 1–4 ml of 20% aqueous methanol, and 1 h extractions. Total cell volumes from monocultures were derived from dry weights by considering typical cell densities of 1 g / ml and water contents of 80% by weight. For mixed cyanobacterium/heterotroph cultures, biovolume partition between partners was estimated from bulk weights and weight partition based on Chl *a* determination, as above.

### Cyanobacterial growth with urea as sole nitrogen source

N-free 50% BG11_0_ medium (50% v/v medium + 50% distilled water) was supplemented with urea to varying concentrations (100, 10, 5, and 2 μM), keeping the total N available constant by modifying medium volumes. For a negative control, 50% BG11_0_, was used, with 50% BG11 used as a positive control (nitrate as N source). Each concentration was assayed in three independent replicate cultures. Prior to testing, *M. vaginatus* and *Synechocystis* sp. were starved of N in 50% BG11_0_ until cessation of visible growth (2 weeks and 1 month, respectively) to deplete internal N, then incubated for 2 weeks under standard conditions before scoring growth visually as positive or negative. *N. punctiforme*, a nitrogen-fixer, will grow in N-free medium, though it prefers exogenous N sources. In this case, we evaluated the concentration of urea at which it switched to diazotrophic growth, by counting the ratio of heterocysts to vegetative cells [42] under each condition.

### Bulk biocrust microbiome growth as a function of urea concentration

A dry biocrust sample weighing 5 g was placed in a 15 ml centrifuge tube and wetted to saturation by soaking overnight in 15 ml of 50% BG11_0_ liquid medium supplemented with 0.5 g/L each of sucrose, glucose, acetate, mannitol, and pyruvate. It was then slurried by vortexing and centrifuged at 9400 xg for 10 min. Five hundred μl was taken from the resulting supernatant, which contained the dislodged microbial community, and used to inoculate 50 ml vented culture flasks containing the same medium. These were further amended with urea to reach from 1 μM to 1 mM urea, in triplicate, then incubated at 23°C without illumination. Growth was monitored by absorbance measurements at 600 nm using a spectrophotometer (UV-1601, Shimadzu Corporation) every 24 h over the course of 10 days, exponential growth rates determined by logarithmic fitting of the time course data. Final absorbance values were used as a relative measure of yield.

### Whole genome sequencing, assembly, and annotation

DNA was extracted using the DNeasy PowerSoil Pro Kit (Qiagen) according to the manufacturer's instructions. For *M. vaginatus*, a well-grown culture was centrifuged into a 1 ml pellet. For *Massilia* sp. METH4, a lawn of colonies from one Petri plate of solid medium was used, after scraping and suspending in the CD1 solution from the kit. The eluted DNA product was digested by RNase and Proteinase K before quantification with a Qubit fluorometer (ThermoFisher). DNA quality was checked, a library prepared, and sequencing performed on the PacBio HIFI platform at the Arizona Genomics Institute (University of Arizona, Tucson). Sequences were assembled using Flye (2.9-b1768). Genomes were annotated using the RAST tool kit using default parameters [43].

### Targeted gene expression analysis of urea-related genes by RT-qPCR

Two ml pellets of well-grown *M. vaginatus* PCC 9802 in C free medium, three Petri plate lawns of *Massilia* sp. METH4 from N-free media, and two pellets of co-cultures in C and N-free medium, were used to extract around five hundred ng of RNA using the RNeasy Mini Kit (Qiagen) following the manufacturer's instructions. cDNA was synthesized using the iScript cDNA Synthesis Kit (Biorad). One μl of the resulting cDNA was used as qPCR template. Transcripts were amplified using primer sets targeting specific genes as described in Table S1, using an ABI 7900 HT Real-time PCR system (Applied Biosystems) and PowerUp SYBR Green Master Mix (Applied Biosystems) according to the manufacturer's protocols (ThermoFisher Scientific). The primers were designed according to the annotated genome sequences from *M. vaginatus* and *Massilia* sp. METH4 using Primer3web version 4.1.0 [44]. Transcript level was quantified in six biological replicates of each condition using the 2^-ΔΔCT^ method with expression normalized to that of the RNase P RNA gene (*rnpB*) in *M. vaginatus* [45] or to that of the glyceraldehyde-3-phosphate dehydrogenase gene in METH4 [46, 47].

### Statistics

We used a Shapiro-Wilcoxon normality test to determine if the data was normally distributed for every data set examined, which was the case unless otherwise stated. A one-way analysis of variance was used to test differences among treatments, followed by Tukey’s post-hoc tests for data sets that were normally distributed. Non-normal data, were analyzed with non-parametric Kruskal-Wallis tests followed by Nemenyi post-hoc tests. These tests were run using the R package stats [48] and DescTools [49]. Statistical analysis for gene expression was performed using the Wilcoxon non-parametric test.

## Results

### Genomic interrogation of mutualistic strains

We probed the genetic capabilities with respect to urea metabolism of representative strains of *M. vaginatus* PCC 9802 and one of its heterotrophic mutualists, *Massilia* sp. METH4. A previous genome sequence of *M. vaginatus* PCC 9802 available in public databases was assembled into several contigs and its completeness was uncertain, so we re-sequenced it using PacBio long-read technology. We selected a random subset of 40 000 reads per strain, which was ~1/15^th^ of the initial set of reads for each strain, and yielded ~280 Mbp of sequence. The quality scores for *M. vaginatus* PCC 9802 and *Massilia* sp. METH4 were classed as Q30, indicating highly accurate long reads with >99.9% single-molecule accuracy.

For *M. vaginatus* PCC 9802, this yielded a single 6.70 Mbp chromosomal contig and one large 21 Kbp plasmid, in which we detected 8016 open reading frames, of which 3430 received functional assignments. During our work (2022), the previously existing genome sequence (Accession No.: PRJNA486193) was updated with a long-read based version, which is essentially identical to ours, except that it lacks the plasmid. The older genome sequence was only used to compare with the current one. A search for homologs of genes involved in urea metabolism found, consistent with a prior analysis run on the old genome version [50], a full complement of homologs coding for standard urease (and its maturation proteins), enzymes for the urea-ornithine cycle (except for arginase), as well as genes for the urea permease system UrtA-E. In addition, homologs for the rarer ATP-dependent urea amidolyase ureolytic system, composed of separately encoded allophanate hydrolase (ah, both subunits; EC 3.5.1.54) and urea carboxylase (uc, both subunits; EC 6.3.4.6), were also present.

The genome of *Massilia* sp. METH4 was assembled to a single contig, 6.88 Mbp long and 66% GC, coding for 6011 open reading frames, of which 3430 could be given functional assignments. Here we found homologs for urease and its accessory proteins, urea permease, and four of the five enzymes in the standard urea cycle, except for arginase. Homologs for alternative arginine metabolizing enzymes like agmatinase were not found either, although a homolog for arginine decarboxylase was present.

Both *M. vaginatus* PCC 9802 and *Massilia* sp. METH4 have well defined repertoires for urea uptake and utilization but homolog similarity between both organisms was of low to moderate (37–74%) excluding horizontal gene transfer as a reason for this.

Both organisms contained homologs for biosynthesis and hydrolysis of cyanophycin, a non-ribosomal polymer of aspartic acid and arginine that serves as a N reserve and may serve as overflow repository to the urea cycle. Although common in cyanobacteria, cyanophycin is rather rare among heterotrophs [51].

### Transcriptomic interrogation of mutualistic strains

**Figure 1:**
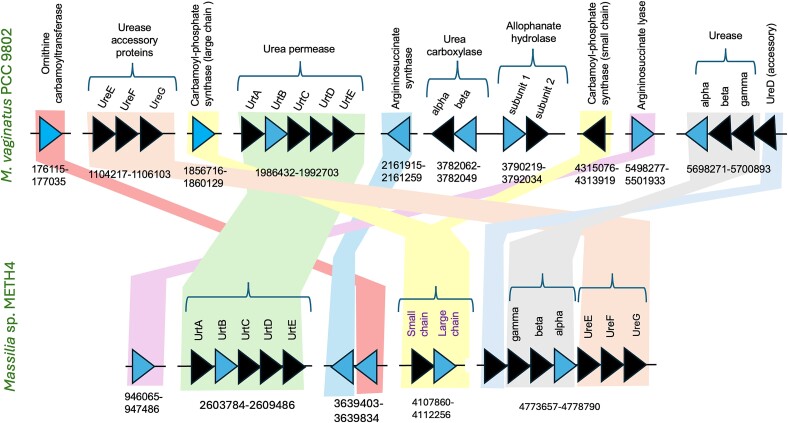
Genes coding for urea-related enzymes in the genomes of *M. vaginatus* PCC 9802 and *Massilia* sp. METH4. Numbers indicate start and end nucleotide positions, arrows the direction of transcription. Genes shaded in blue were targeted for transcriptomics.

**Figure 2:**
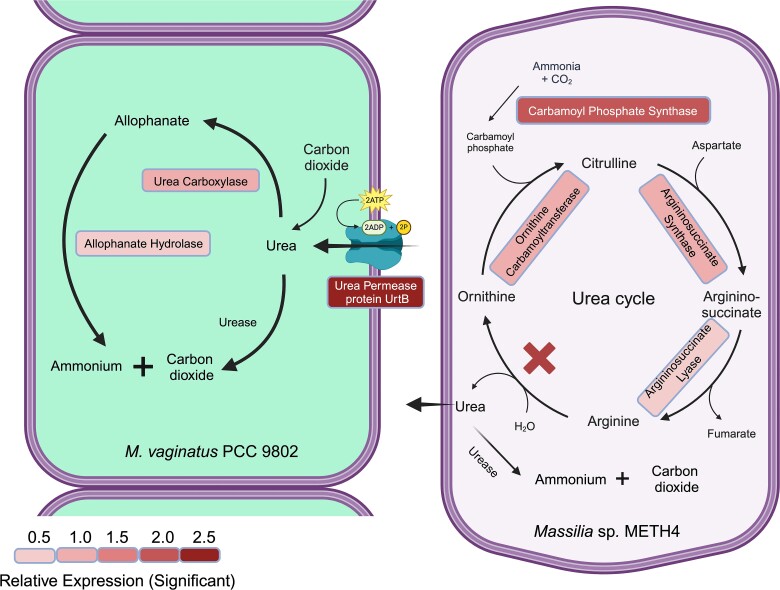
Genes upregulated in mutualist C and N-free co-cultures of *M. vaginatus* PCC 9802 and *Massilia* sp. METH4. Comparisons are to C free, N replete monocultures of the cyanobacterium and to N-free and C containing monocultures of the heterotroph, respectively. Boxes next to specific genes are color coded according to its relative log-fold expression over monoculture conditions and are only shown when changes were statistically significant (*n* = 6; Wilcoxon test *P* < 0.05). Full datasets are presented in Figs. S1 and S2.

Based on the genomic information above, we determined differential transcription of selected genes to represent the major functions related to urea metabolism by RT-qPCR. The genes analyzed are shaded in blue in Fig. 1, the function of each enzyme is included in Table 1, and the primers used are in Table S1. They were selected to target at least one gene in each contiguous set, ensuring to also target every urea-related gene outside apparent operons. To gauge the influence of partner interactions, we assessed levels of transcription under three conditions: (1) *M. vaginatus* in monoculture, in N replete and C free medium, (2) *Massilia* sp. METH4 in monoculture in N-free supplemented with sucrose as C source, and (3) *M. vaginatus* and *Massilia* sp. METH4 in co-culture on N and C free media, making their growth dependent on mutualistic exchanges. Gene expression levels of *M. vaginatus* genes in (3) are reported as their ratios to levels assessed in (1), and levels in *Massilia* sp. METH4 in (3) in relation to those found in (2). The results of these determinations are condensed in Fig. 2, with detailed data and statistics extended in Figs. S1 and S2. When growing mutualistically, *M. vaginatus* significantly upregulated (*P* < 0.05) both enzymes in the allophanate hydrolase-urea carboxylase system as well as the urea permease system (gauged by UrtB). No significant changes in relative expression were detected for either the ATP-independent urease or any urea cycle genes. This suggests that in co-culture *M. vaginatus* took up urea, obtaining ammonium through the uc/ah system. Under mutualistic growth, *Massilia* sp. METH4 upregulated (*P* < 0.05) the expression of all four urea cycle genes tested from levels found in N-free monoculture, but we found no differential expression of either its urease or its urea permease system. This suggests that mutualistic growth enhances the production of urea in the heterotroph, but not its recovery from the medium nor its hydrolysis to ammonium, even when, under both conditions, growth is diazotrophic. Overall, these results are consistent with the notion that urea is likely the vehicle for interspecies N transfer between these two organisms.

**Table 1:** Function of enzymes related to urea synthesis and utilization in *Microcoleus vaginatus* PCC 9802 and *Massilia* sp. METH4.

**Organism**	**Enzyme**	**Function**
PCC 9802	Urea Carboxylase	Carboxylates urea to allophanate
PCC 9802	Allophanate Hydrolase	Hydrolyzes allophanate to ammonium and carbon dioxide
PCC 9802, METH4	Urea Permease Protein UrtB	Part of the urea uptake system
PCC 9802, METH4	Urease	Hydrolyzes urea into ammonia and carbon dioxide
PCC9802, METH4	Carbamoyl Phosphate Synthase	ATP-dependent synthesis of carbamoyl phosphate from ammonia and bicarbonate
PCC9802, METH4	Argininosuccinate Synthase	Synthesizes argininosuccinate from citrulline with aspartate
PCC 9802, METH4	Argininosuccinate Lyase	Hydrolyzes argininosuccinic acid to arginine and fumarate
PCC 9802, METH4	Ornithine Carbamoyl transferase	Synthesizes citrulline from carbamoyl phosphate and ornithine

### Physiological interrogation of mutualistic strains

**Figure 3:**
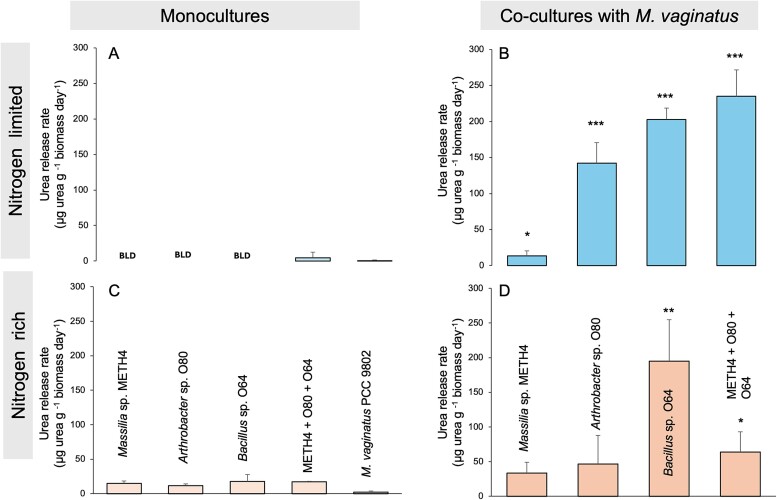
Rate of urea release to the medium calculated in culture under different growth conditions. Averages of triplicates are plotted with error bars representing the standard deviations. Panels a and C are for monocultures or mixtures of heterotrophs. Panels B and D are for co-cultures of heterotrophs with *M. vaginatus*. Panels a, B are for media free of nitrogen, and panels C, D for media containing nitrate-N. The biomass used for co-cultures includes both photo- and heterotrophs. BLD indicates concentrations below the limit of detection, in this case <2 μM. (^*^) on top of the bars of panels B and D represent statistical differences between the urea detected in the monoculture medium and that of its corresponding co-culture with *M. vaginatus* PCC 9802*.*  ^*^ represent a *P* < 0.05, ^**^ represent *P* < 0.01, and ^***^ represent *P* < 0.005.. Full set of concentration data and calculations are presented in Table S2.

In light of the results above, demonstrating that our mutualistic heterotrophs release urea would be required to support its role as N-carrier, particularly as the urea production by bacteria has been only rarely reported, and only few bacteria demonstrably produce and excrete urea in culture [52], although pertinent studies are few. For this, we tested three previously isolated strains that can demonstrably support mutualism with *M. vaginatus* [19]. When heterotrophs were cultured independently in an N-free medium, no urea was detected in the medium, and hence any rates of release must have been extremely low (Fig. 3A; Table S2). When these three isolates were cultured together, urea could be detected, but the biomass-specific rate of release was still very low. Intracellular urea concentrations under the same conditions (measured in a separate experiment) were around or just below 4 mM depending on strain (Table S2), speaking for an effective transport system that maintains concentration gradients of close to three orders of magnitude across the cell membrane, even when urea is quite permeable across it [53, 54]. By comparison, levels of extracellular ammonium in these cultures were also extremely low or undetectable, and intracellular levels, if detectable, in the low μM range (Table S3). When N-limited conditions were used to grow the isolates in co-culture with *M. vaginatus* (Fig. 3 B), however, there was an increase in the rate of urea released in all strains, differences attaining high significance for each heterotroph co-culture (with vs. without the cyanobacterium, *P* < 0.017). Rigorously, that the origin of extracellular urea detected in the medium of co-cultures came from the various heterotrophs and not from the cyanobacterium is not absolutely certain, and that is why we report rates on the basis of total co-culture biomass (Fig. 3). However, such a scenario would collide with the transcriptomic evidence in which the urease cycle was not upregulated in *M. vaginatus* PCC 9802 when growing mutualistically, but it was in *Massilia* sp. METH4. *M. vaginatus* urea release rates in isolation (both under N-starvation or growing in N-replete medium; Fig. 3) were not different from zero. In view of these two findings, a urea source in the phototroph in co-cultures seems unlikely. We conclude that the presence of the cyanobacterium elicited the controlled release of urea to the medium in the heterotrophs, noting that release rates if calculated on the basis of heterotrophic biomass alone would have been about three-fold higher, because biomass of heterotrophs in N-limited co-cultures hovered around 35% of total (see Supplementary Extended Data file).

For comparison, when the isolates were cultured under N replete conditions (Fig. 3C), urea release could be detected in all, though only at low biomass-specific rates. Intracellular concentrations of urea under these conditions ranged between 7 and 17 mM, higher than those found under N limitation. Again here, all release rates increased significantly when the strains were co-cultured with the cyanobacterium (*P* < 0.108), confirming the release enhancement by the presence of *M. vaginatus,* even under N-replete conditions, in which mutualistic exchanges are not necessary for the cyanobacterium.

**Figure 4:**
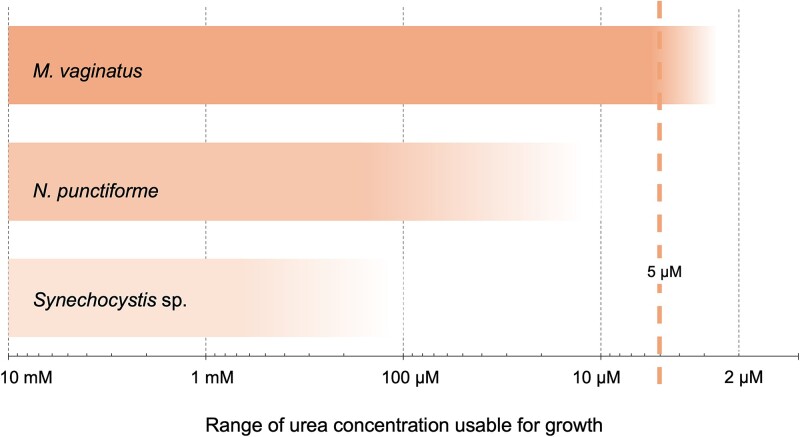
Cyanobacterial growth as a function of urea concentration. Gray dotted lines represent concentrations of urea tested. Solid bars denote the range where growth supported by urea was observed (*n* = 3), the fading region includes the region of uncertainty. Orange dotted line marks the lowest concentration for growth in *M. Vaginatus* (and overall). For *Nostoc,* the range shown corresponds to non-diazotrophic growth as determined separately (Fig. S3).

On the side of the phototroph and given its reported preference for urea when provided at high concentration [28], we wanted to obtain evidence of its ability to obtain and use it in a range of concentrations that may be more relevant to the mutualistic system in the laboratory model, as well as in the natural habitat (see next section). For this, we assayed qualitatively its capacity to grow on unlimited external urea at widely varying concentrations (Fig. 4). We could detect consistent (*n* = 3) growth of strain PCC 9802 down to 5 μm, but not 2 μM urea. To place these in context, we also tested two additional cyanobacterial strains used commonly as experimental models. The lower limit for growth of *Synechocystis* sp. PCC 6803 was 1 mM, one-hundred-fold higher than that of *M. vaginatus. N. punctiforme ATCC 29133,* a heterocystous nitrogen fixer, switched from urea-based growth to diazotrophic growth between 10 and 100 μM urea, judging from a sharp increase in the frequency of heterocysts (Fig. S3), indicating that at lower concentrations its urea metabolizing capacity failed to sufficiently support growth. This confirms that *M. vaginatus* is indeed comparatively well-equipped to use urea as a N-source, even at very low concentrations.

### Urea availability and responses in the natural biocrust environment

**Figure 5:**
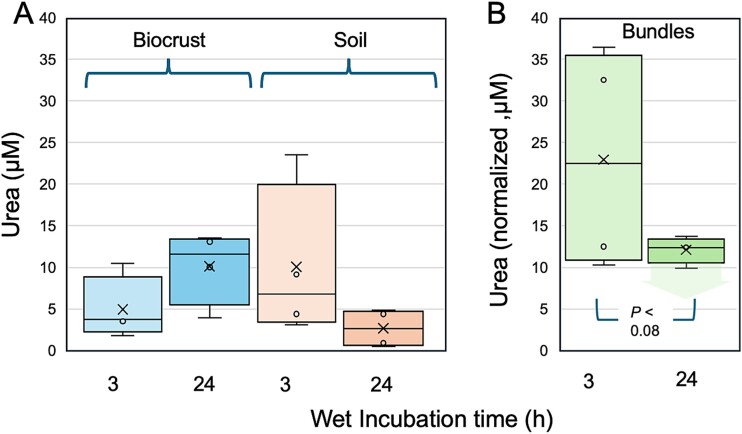
Pools and dynamics of urea in biocrust domains during short (3 h) and long (24 h) incubations. Data represent four independent tests. (A) Direct determinations of concentration in the soil solution of bulk biocrust proper (biocrust) or soils immediately below biocrusts (soil). Mean differences in time or among sample types were not significant (*P* > 0.2). (B) Concentrations attained in collections of native bundles (averaging 47 bundles per collection) excised from biocrusts and incubated separately. Concentrations are normalized to the total bundle biovolume in each sample, by adjusting measured concentrations to an incubation volume equivalent to 1000-fold the bundle biovolume present. Urea in the 24 h incubations was below detection level in the unnormalized assay, but the normalized detection levels have been preserved in the graph, as a highest-case scenario for comparisons with the 3 h incubations, which was significant with a T-test. Detailed datasets and calculations can be found in Table S4.

Urea concentrations in the soil solution at water saturation in recently wetted (i.e. activated) native biocrusts, although variable, were consistently around 5 μM, and those of the soils below the bounds of the crust somewhat higher, around 10 μM (Fig. 5), though not significantly different (*P* > 0.21). These are comparable to reported contents of native (non-fertilized) temperate grassland and agricultural soils, which contain 20–90 ng-N g^−1^ urea [55], roughly translating to 7–32 μM in the soil solution at water saturation. We found no statistically significant differences in either pool with incubation duration under standard conditions for phototrophic cultures (*P* > 0.45).

An assessment of extracellular urea concentration in collections of *M. vaginatus* bundles excised directly from native biocrust carrying its attached cyanosphere after standard laboratory incubations painted a different picture (Fig. 5 B and Table S4). In the short term (3 h), urea (normalized to a common 1000-fold level of biovolume of bundles to volume of medium) reached 6–30 μM. This would correspond to urea concentrations of several hundred μM if one considered a more typical *in situ* situation with a cylindrical volume of influence 100 μm-wide around each bundle. However, longer incubations (24 h) resulted in a drop of urea below detection, which was significant even assuming a worst-case scenario in which all 24 h concentrations were just at the detection level (T-test, one sided, non-paired; *P* < 0.08). Mutualistic assemblages of *M. vaginatus* and its cyanosphere are thus dynamic hotspots of urea in the context of the biocrust microbiome.

**Figure 6:**
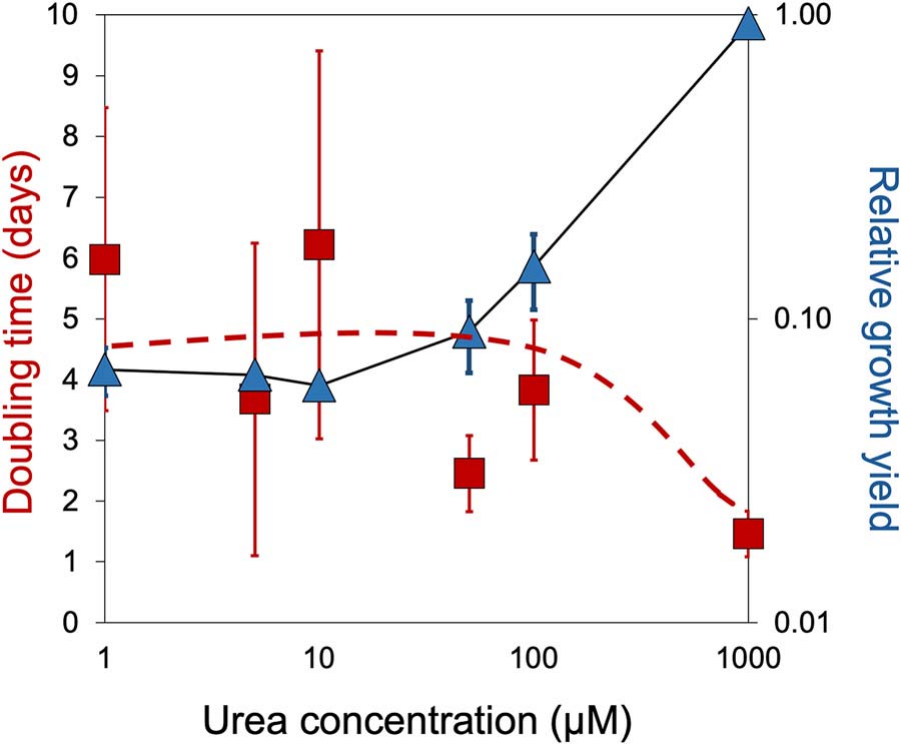
Growth performance of enrichment cultures of the bulk biocrust microbiome as a function of urea concentration provided. Squares represent doubling times and triangles represent relative growth yields after 10 days. Error bars show the standard deviation of *n* = 3 independent cultures.

We then tested the ability of native biocrust heterotrophs to grow as a function of urea in enrichment cultures in liquid N-free media supplied with (i) varying concentrations of urea and (ii) a varied mixture of C sources. These were inoculated with bacterial slurries prepared from bulk biocrust samples, growth rates and final yields determined by turbidity. Growth rates and yield in the controls without urea reflect contributions by diazotrophs, and any increases above this level represent added contributions by microbial growth on the urea provided. We found a biphasic trend with urea concentration for both growth rates and yields (Fig. 6). The final growth yield of enrichments depended directly on the amount of urea provided only above the 10^−5^ M range, essentially being constant below it to the inclusion of cultures with no urea added. Thus, urea could apparently contribute significantly to overall growth only above this inflection point. Growth rates, although more variable, were consistent with this interpretation, doubling times trending down from those with no urea only well above 10 μM. These results suggest that most heterotrophs in the biocrust microbiome are unable to effectively use the existing urea pools in the bulk soil as a source of N.

**Figure 7:**
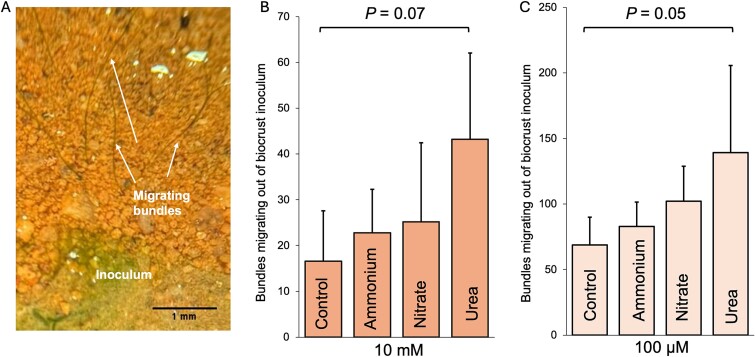
Effect of urea addition on bundle migration away from established biocrusts. (A) Photomicrograph showing the principle of the bundle migration assay. (B) Analytes (shown in bars) added as 10 mM solutions. (C) Analytes added as 100 μM solutions. Columns represent the average of five independent assays and the error bars represent the standard deviations.

It was previously shown that the addition of usable N sources (nitrate, ammonium) to *M. vaginatus* bundles carrying their cyanosphere, altered motility responses that keep them together by weakening their interdependence [28]. We thus tested if urea could also elicit such an effect in natural assemblages using a bundle migration assay that quantifies the tendency of the cyanobacterium to migrate away from its established spatial organization in native biocrusts (Fig. 7). We could observe that provision of urea elicited a statistically significant enhancement over controls in the bundle migration frequency into crustless soil, as did the provision of nitrate or ammonium. Although urea was most efficient among the three sources provided, differences between sources were not significant. Hence, provision of external urea also relieves microbial behavior geared towards maintaining cyanobacteria and its heterotrophic mutualists in close proximity.

## Discussion

### Urea as an interspecies N carrier

As a whole, the results presented here speak to a central role of urea in the transfer of N within the *M. vaginatus* / cyanosphere symbiosis, a mutualistic association that is foundational for the development and recovery of biocrusts.

The genetic potential and gene regulatory patterns ascertained using cultivated representative isolates are consistent with the view that urea is synthesized in the heterotrophic partner specifically in the presence of the cyanobacterium, and that the cyanobacterium in turn poises its metabolism for urea uptake and its intracellular hydrolysis (Fig. 2). In this regard, the absence of a canonical arginase homolog in the heterotroph may indicate the presence of a somewhat unconventional urea-ornithine cycle, but we do not see it as unusual. In fact, there is some precedence for this: in *Synechocystis* sp. PCC 6803, e.g. a standard arginase homolog is absent, even when the corresponding activity can be demonstrated biochemically [56]. In the reconstruction of metabolic pathways from genomes, the “missing gene” problem is pervasive [57, 58]. It stems from imposing pathways that fit the known traits and steps of a few characterized model organisms, a fact that underestimates biochemical diversity in nature. Arginases are indeed rare in complete genomes of *Massilia* sp. available at NCBI (only 2 of 24, in our analyses).

Additionally, physiological experiments with cyanosphere heterotrophic cultures showed the controlled extracellular release of urea in the presence of the cyanobacterium, but not otherwise, even when they grow diazotrophically (Fig. 3), concentrations in the medium reaching 10^−4^ − 10^−5^ M and intracellular loads reaching 7–17 mM (Table S2). The release of N-rich urea is quite rare among bacteria, and it does not make sense on purely metabolic economy grounds, particularly under N-limitation; it could be of fitness value only in the context of a cross-feeding mutualism [59].

*M. vaginatus* appears unusually proficient in growing on urea as sole N source down to concentrations in the several micromolar range (Figs. 4 and 5) at least compared to other cyanobacteria (Fig. 4) and to the bulk of bacteria in the biocrust microbiome (Fig. 6). It should theoretically be able to take advantage of any of the urea pools detected in the various biocrust compartments (Fig. 6). As Urt-type bacterial urea uptake systems show affinity constants in the range 10–30 μM [60] (that of *Synechocystis* sp. PCC 6803 being even lower around 1 μM; [61]), the differential lack of growth at low concentrations is best ascribed to differences in internal metabolic processing capacity. Finally, *M. vaginatus* motility behavior assessed in native populations is significantly altered by the provision of external urea, resulting in a weakening of controls that keep it close to established mutualisms (Fig. 7).

### How can urea transfer contribute to partner specificity?

The choice of N carrier seems logical in that urea has a high N:C molar ratio and can be directly linked to cyanophycin stores through arginine in the urea cycle, in either producer or consumer. The fact that it is unparalleled among known C for N symbioses, may well indicate that it contributes uniquely to attaining partner specificity. In this regard, keeping extracellular concentrations low seems key, as we found in the field measurements of bulk soils, and long incubations of excised natural bundles. Such low micromolar urea pools are out of range for effective action of extracellular soil ureases, known to have affinity constants solidly in the mM range [62–65]. Furthermore, extant heterotrophs in the bulk microbiome demonstrably had difficulty growing better than diazotrophs at those concentrations. In short, extracellular urea at or below the 10 μM mark should remain “under the radar” of potentially adventitious cheaters in the microbiome (Figs. 4 and 5) or of competing cyanobacteria.

It is tempting to ascribe an important role in these effects to the ATP dependent uc/ah system, not only because it is upregulated in *M. vaginatus* under mutualistic growth, but also because of its rarity, and because it requires ATP expenditure compared to standard urease. In a genomic survey conducted in 2019 [50], less than 3% of cyanobacterial genomes contained the uc/ah (compared to the 76% that contained a complete set of genes for urease and its maturation proteins). However, assigning a mechanism is not as straightforward. The alternative intracellular ureolytic systems appear to have affinities clustering at different urea concentrations: K_m_ values for ureases in various organisms, including cyanobacteria, range widely from 10^−5^ to 10^−2^ M, most clustering around 10^−4^–10^−3^ M [60, 66–69]. A few determinations available for the ATP-dependent system tend to be lower, around 10^−5^ M [70–72]. It is thus plausible that the ATP-dependent system outdoes standard urease on this basis, though in the absence of direct characterizations of the actual *M. vaginatus* enzymes this would need confirmation. Alternatively, the use of urease may be differentially impeded by a lack of its metal cofactor, Ni, which is extremely scarce in biocrusts compared to other metals [73]. In this scenario, a Ni-free enzyme system may provide an advantage (even if at the cost of additional energy for urea hydrolysis).

### Urea hot spots and hot moments in biocrusts

Unfortunately, the biogeochemistry and microbiology of soil urea, except perhaps in agricultural settings under urea fertilization [74–76], has not been studied with any detail to provide a wider context for our findings. It is not even mentioned in comprehensive reviews of N cycling in biocrusts [17], arid soils [77] or even soils at large [78]. Our necessarily moderate first assessment in biocrusts suggests that soil solution pools of bulk biocrusts are low enough to be beyond the reach of typical soil ureases, with no significant temporal dynamics. But they show also that urea is a dynamic and very significant component of N cycling around their foundational *Microcoleus /* cyanosphere mutualisms, which can be considered urea hotspots (at least during initial hot moments after wetting). This adds to the spatial complexity of chemical–physical microenvironments in biocrust [79], including those involving other N-cycling activities and pools [80], highlighting the need for microscale assessments to explain their emergent properties.

### Potential applications

Our findings may have immediate potential applications in cyanobacterial biocrust restoration. Biocrust inoculum supplied to bare soils generally suffers from high losses or low viability [81–83], in spite of technical improvements that address perceived hindrances, such as the provision of biocrust probiotics [20], the avoidance of pests [84], or the physiological conditioning of inoculum to harsh conditions [85]. Yet some of these losses can be ascribed to more mundane reasons, like removal of inoculum particles on the soil surface by wind entrainment before cyanobacteria effectively migrate into the surrounding bare soil. Our results show that providing urea during a first wetting could enhance this migration, coaxing bundles to anchor inoculum particles to the surrounding soils (Fig. 7). Experiments to test the benefits of such an approach are currently underway.

## Supplementary Material

Supplementary_information_wrae246

Supplementary_Data_File_wrae246

TableS4_wrae246

## Data Availability

The sequencing data generated in this study have been deposited in the NCBI database under the BioProject PRJNA1078857 and BioProject PRJNA1078815 respectively. All other raw data generated in this study and not directly given in Supplementary Tables are provided under Supplementary Data.
