# Supplementary material for: Urea-based mutualistic transfer of nitrogen in biological soil crusts
Source: ISME J. 2024 Dec 13;19(1):wrae246. doi: 10.1093/ismejo/wrae246 (PMC11844795; doi:10.1093/ismejo/wrae246)

SUPPLEMENTARY FIGURES AND TABLES


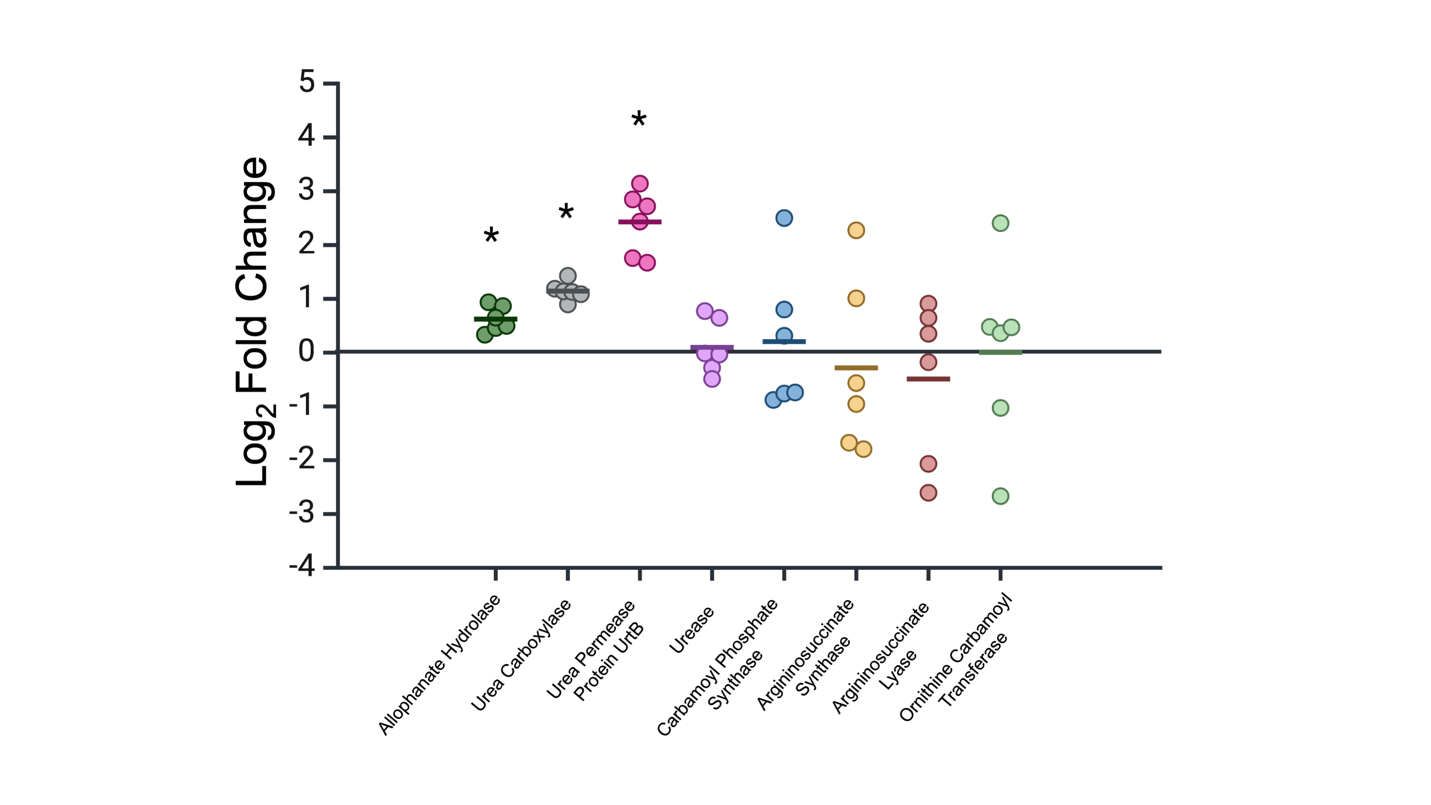


Figure S1: Relative expression of urea-related genes in *M.vaginatus* PCC 9802 determined by RT-qPCR. Genes with significant (*P*<0.05; Wilcoxon signed rank test) overexpression in *M.vaginatus* PCC 9802 co-culture with *Massilia* sp. METH4, compared with monoculture in N-replete medium are marked with asterisks. *n* = 6 independent cultures. Expression in each condition normalized to that of *rnpB*.


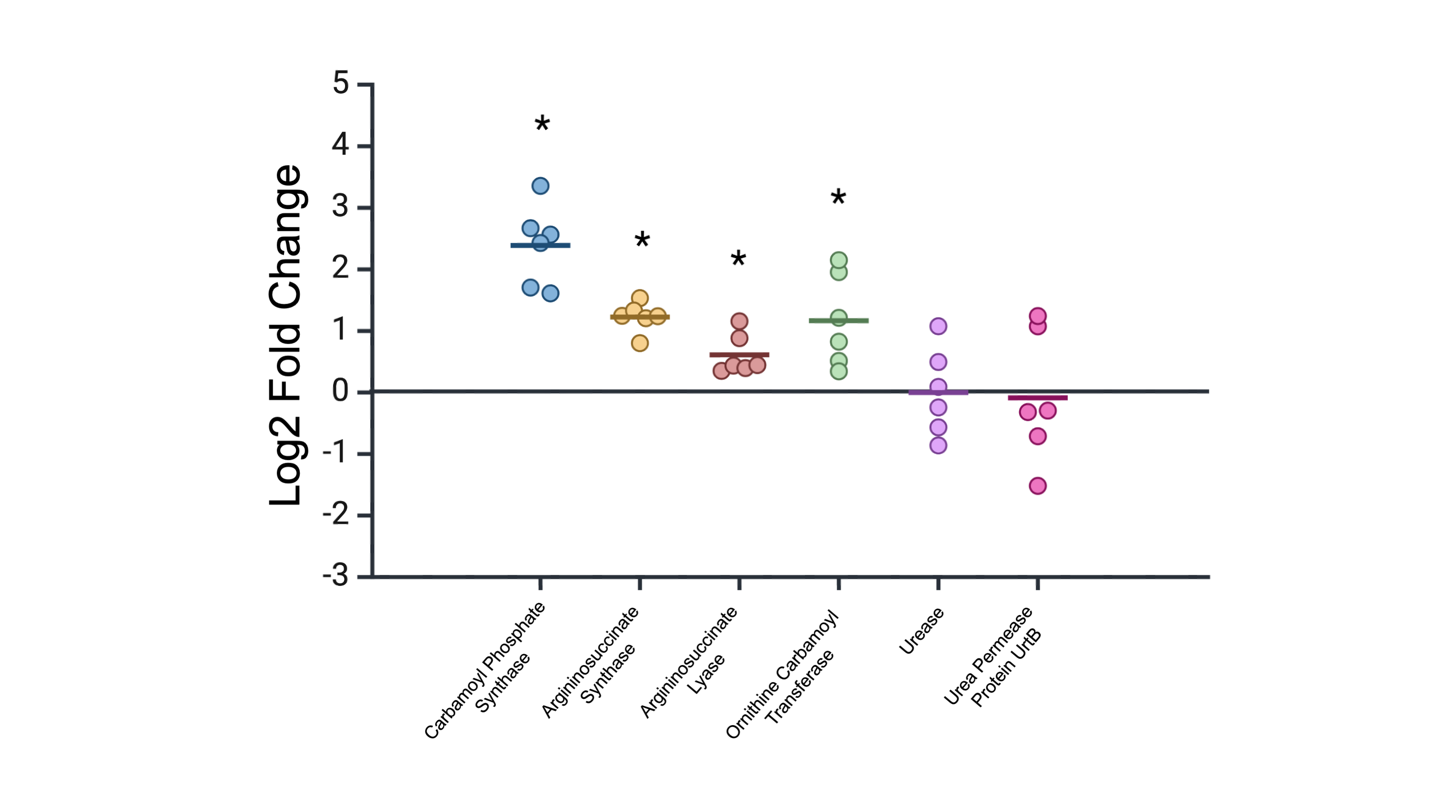


Figure S2: Relative expression of urea-related genes in *Massilia* sp. METH4. Genes that are significantly upregulated (P<0.05; Wilcoxon signed rank test) in co-culture with *M.vaginatus* PCC 9802 compared with monoculture under C-replete, N-free conditions are marked with an asterisk. *n* = 6 independent cultures. Expression levels under each condition are normalized to that of GAPDH.


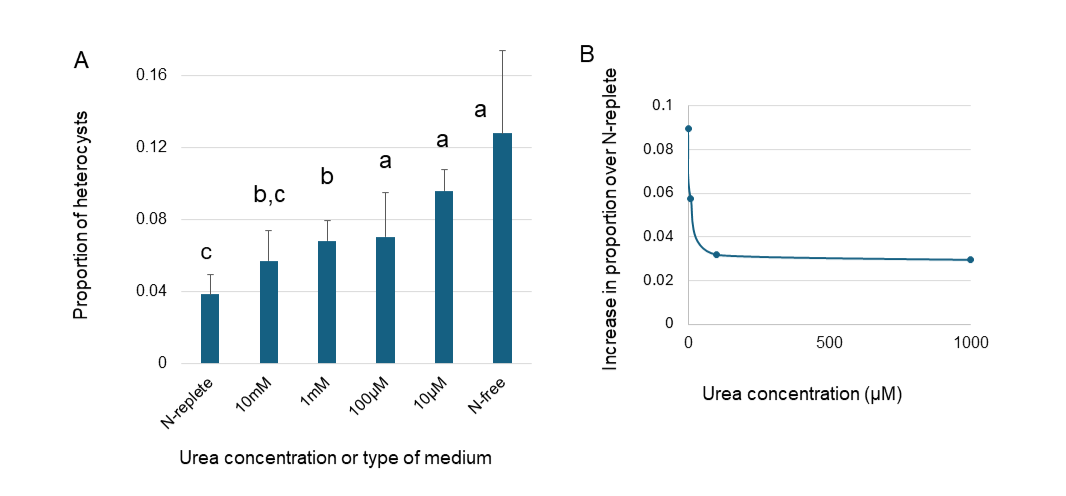


Figure S3. Proportion of heterocysts to vegetative cells in *Nostoc puctiforme* ATCC 29133 under various concentrations of urea. A: Proportions under various concentrations of urea (*n* = 4), inclusive N-free (BG11_0_), and N-replete (BG11) media. Same letter indicates proportions not significantly different according to T-tests (*P* <0.05). B: Increase in mean proportion of heterocyst over mean levels in N-replete media showing rapid increase between 100 and 10 µM (10 mM treatment excluded for clarity).

Table S1. Primers used in RT-qPCR for the determination of transcript levels of query genes by organism.


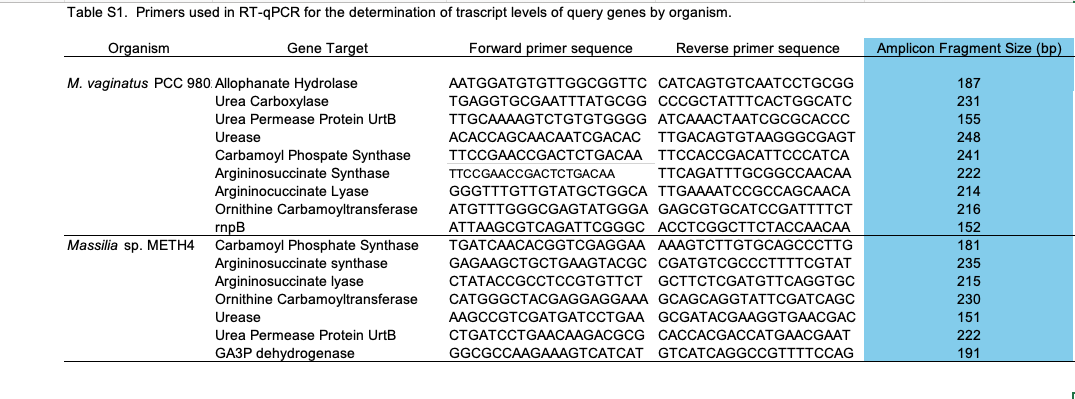


Table S2. Intra- and extracellular concentrations of urea attained in cultures or co-cultures of mutualists (µM). Means of 3 replicate cultures for extracellular concentrations (± SD), where "bdl" stands for below detection limit, in this case <2 µM. Intracellular concentrations are means of two independent determinations.


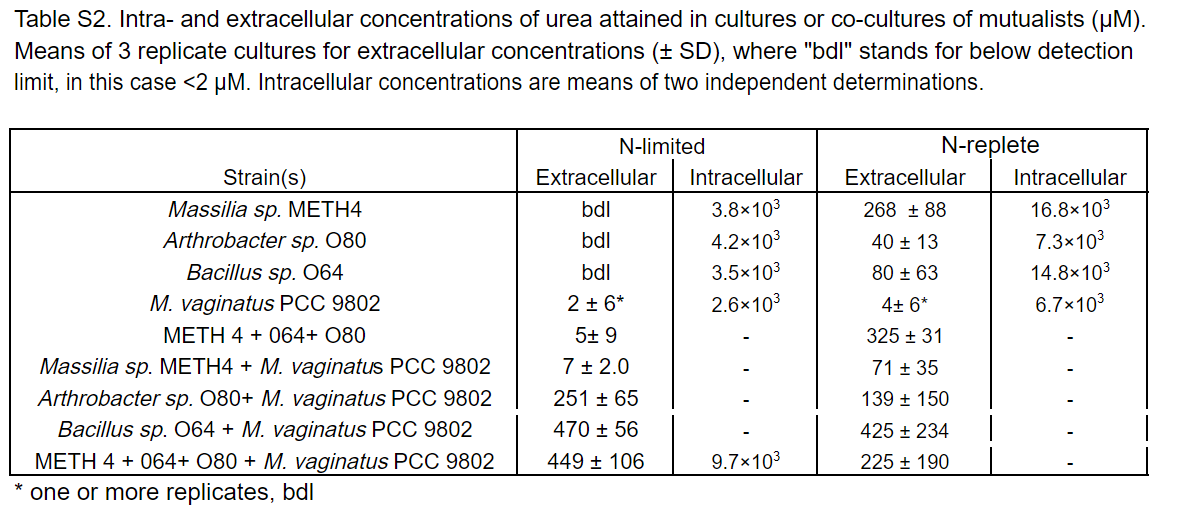


Table S3. Intra- and extracellular concentrations (µM) of ammonium attained N-free cultures of mutualistic strains. Means of duplicate cultures are given, where bdl stands for below limit of detection.


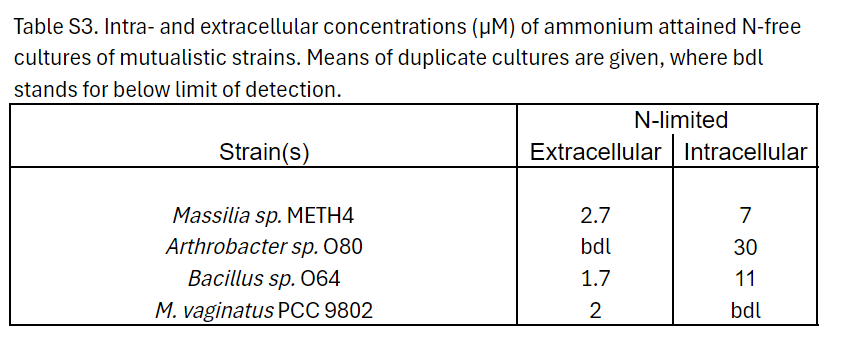

Supplement: Supplementary_information_wrae246 [file supplementary_information_wrae246.docx]
